# Epigenetic silencing in transgenic plants

**DOI:** 10.3389/fpls.2015.00693

**Published:** 2015-09-10

**Authors:** Sarma Rajeevkumar, Pushpanathan Anunanthini, Ramalingam Sathishkumar

**Affiliations:** ^1^Molecular Plant Biology and Biotechnology Division, Central Institute of Medicinal and Aromatic Plants Research Centre, BangaloreIndia; ^2^Plant Genetic Engineering Laboratory, Department of Biotechnology, Bharathiar University, CoimbatoreIndia

**Keywords:** homology-dependent gene silencing, post-transcriptional gene silencing, systematic acquired silencing, transcriptional gene silencing, transgenic plants

## Abstract

Epigenetic silencing is a natural phenomenon in which the expression of genes is regulated through modifications of DNA, RNA, or histone proteins. It is a mechanism for defending host genomes against the effects of transposable elements and viral infection, and acts as a modulator of expression of duplicated gene family members and as a silencer of transgenes. A major breakthrough in understanding the mechanism of epigenetic silencing was the discovery of silencing in transgenic tobacco plants due to the interaction between two homologous promoters. The molecular mechanism of epigenetic mechanism is highly complicated and it is not completely understood yet. Two different molecular routes have been proposed for this, that is, transcriptional gene silencing, which is associated with heavy methylation of promoter regions and blocks the transcription of transgenes, and post-transcriptional gene silencing (PTGS), the basic mechanism is degradation of the cytosolic mRNA of transgenes or endogenous genes. Undesired transgene silencing is of major concern in the transgenic technologies used in crop improvement. A complete understanding of this phenomenon will be very useful for transgenic applications, where silencing of specific genes is required. The current status of epigenetic silencing in transgenic technology is discussed and summarized in this mini-review.

## Introduction

Conventionally closely related species are easier to breed than inter species/genus due to compatibility issues, and this is considered a major limitation. Transgenic technologies have allowed gene transfer to completely unrelated organisms. All these advances have increased the global transgenic plant cultivation to 181 million hectares (James, 2014). Transgenic plants with stacked genes are gaining more importance lately. Here, different genes are expressed in one transgenic plant from a single transformation event, or in consecutive steps either by re-transformation or by conventional genetic crosses involving different transgenic lines expressing a single transgenic event (Dietz-Pfeilstetter, 2010). To date, diverse traits such as disease resistance, stress tolerance, nutritional improvement, and the use of plants as host systems to produce economically important molecules have been successfully proven (Ahmad et al., 2012). The purpose of gene transfer to plants in all the above cases was to achieve specific desirable traits, where lines that failed to meet expectations are discarded, so that the best performers can be propagated (Kohli et al., 2006, 2010). Initial reports of unforeseen low gene-expression levels or silencing of transgenes were considered failures. Later, those minor glitches emerged as a principal factor elucidating the role of epigenetics in this emerging technology (Meyer et al., 2013).

A major prerequisite for plant expressing a transgene is stability and segregation. Several reports have documented a deviation from the Mendelian segregation ratios in transgenic plants (Shrawat et al., 2007; Weinhold et al., 2013). This revealed the existence of hitherto unknown cellular mechanisms which regulate expression of transgenes. In the last three decades, many reports on transgene instabilities as well as the reasons behind these events were the main focus (Charrier et al., 2000; Graham et al., 2011; Stroud et al., 2013). The explanation for inactivation/silencing of transgene activity was a lack of transcription due to methylation of the promoter along with condensation of chromatin, or degradation of transcripts by different mechanism (Fagard and Vaucheret, 2000; **Table 1**).

**Table 1:** Reports of epigenetic silencing in transgenic plants.

Target plant	Gene (s)	Transgene effects	Reference
*Arabidopsis thaliana*	Selectable marker genes (*npt*/*hpt*)	Repeated sequence of target gene at same loci lead to repeat-induced gene silencing (RIGS).	Assaad et al., 1993
*Nicotiana tabacum*	Selectable marker gene (*npt*)	*De novo* methylation mediated silencing of *nptII*	Ingelbrecht et al., 1994
*Petunia hybrida*	Flavonoid hydroxylase gene, maize *A1* gene	Hypermethylation of 35S promoter directed *A1* gene expression lead to variegated flower pigmentation in transgenic *Petunia* lines	Meyer et al., 1994
*Avena sativa*	*bar* and *gusA*	Direct DNA–DNA interaction between multiple transgene copies resulted in silencing of *bar/gusA* gene to different levels.	Pawlowski and Somers, 1998
*Oryza sativa*	*bar* gene	Methylation of Ubi1 promoter lead to silencing of *bar* gene and bialaphos sensitivity in transgenic rice	Kumpatla and Hall, 1998
*Saccharum officinarum*	sorghum mosaic potyvirus strain SCH coat protein (CP) gene	Reduced transcript level lead to post-transcriptional gene silencing (PTGS) of CP gene in transgenic sugarcane.	Ingelbrecht et al., 1999
*Oryza sativa*	*GUS* gene	Reintroduction of GUS gene in GUS transformed rice lead to suppression of GUS expression due to PTGS	Kanno et al., 2000
*N. tabacum*	*GUS* gene	Gene silencing through DNA methylation lead to reduced expression of *GUS* gene in transgenic tobacco lines	Day et al., 2000
*Petunia*	*CHS gene*	White-flowering phenotype due to chalcone synthase transgene-induced silencing as a result of altered methylation in promoter	Kanazawa et al., 2007
*A. thaliana*	Phytochrome A/ DNA methyl transferase I gene	Exonic methylation can lead to chromatin modification further resulting in altered gene expression mediated through reduction in the transcription rate.	Chawla et al., 2007
*N. tabacum*	*nptII*	Target gene was silenced by PTGS based on the loci of intergration	Khaitova et al., 2011
*Gentiana verna*	CaMV35S promoter	*De novo* methylation of the enhancer region of CaMV 35S promoter silencing is triggered by histone H3 deacetylation.	Yamasaki et al., 2011
*A. thaliana*	*A. thaliana* repressor of silencing1 mutant	Mutants treated with sulfamethazine exhibited reduced levels of DNA methylation and released transgene silencing. Exogenous application of p-Aminobenzoic acid restored transcriptional gene silencing (TGS) in SMZ-treated mutants	Zhang et al., 2012
*N. tabacum*	CaMV35S promoter	DNA methylation and heterochromatic histone marks were studied in different epialleles of 35S promoter driven tobacco transgenic calli. Transient loss of euchromatin modifications lead to *de novo* DNA methylation further leading to formation of stable repressed epialleles with recovered eukaryotic marks	Křížová et al., 2013
*N. tabacum*	*A. thaliana* a repressor of silencing gene (ROS1)	Transgenic lines over-expressing *At ROS1*showed higher level of demethylation in promoter as well as coding region of various genes involved in flavanoid biosynthesis and antioxidant defense response	Bharti et al., 2015

## Epigenetics

The British developmental biologist Conrad H. Waddington coined the term “epigenetics”. Epigenetics deals with studies related to interactions of genes and their products, which determine the phenotype of a system (Waddington, 1942). During the course of an organism’s development, cell fate is determined by genes and by other (epigenetic) factors, which underlies the notion of “epigenesis”. Modern biology has redefined as a phenomenon in which a gene’s activity is modulated by modifications of nucleic acids or the physical packaging of the chromatin in which it is embedded.

Two main classes of transgene-silencing phenomena have been reported to date. The first concerns position effects, in which the expression of a foreign gene is negatively regulated by flanking host DNA or chromosomal location (Matzke et al., 2000). The expression of a gene integrated into a region of euchromatin is also influenced by regulatory sequences of host genes (Kohli et al., 2006). Transgene integration into heterochromatic regions also leads to silencing (Grewal and Elgin, 2002).

**FIGURE 1:**
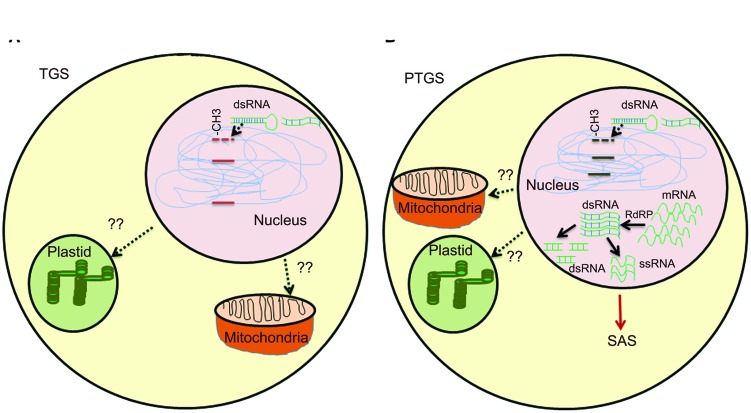
**Overview of gene silencing in transgenic plants. (A)** Transcriptional gene silencing (TGS)- DNA methylation induces dsRNA by endogenous gene or multiple copies of transgenes. Presence of multiple copies of transgene induces formation of dsRNA. Single copy transgene loci could also lead to formation of dsRNA due to high RNA turnover. Methylation of CG, CNG, or CNN region in promoter by different methyltransefrases that leads to TGS. Methylation in heterochromatin region also lead to TGS. T-DNA with transgene integrated as direct or inverted (IR) repeats are inactivated by DNA methylation. Cruciform structures formed by IRs act as substrate for DNA methyltransferases. **(B)** Post-transcriptional gene silencing (PTGS)- Methylation in coding region and high RNA turn over lead to production of dsRNA, abbrreant RNAs, cRNAs. RdRP uses these aberrant RNAs as templates and convert them into a double-stranded RNA, which is further degraded by different dsRNases yielding small dsRNAs and/or ssRNAs. The ssRNAs and/or dsRNAs act as systemic silencing signals, which are transported all over the plant and trigger PTGS in adjacent cells. SAS in mitochondria and plastids are still under study.

The second class of silencing phenomena is based on epigenetic regulation and is a type of inactivation mechanism that can arise when multiple copies of the same or homologous sequence are introduced in a genome. Since interactions between homologous nucleic acid sequences are responsible for these silencing, it is also called homology-dependent gene silencing (HDGS) (Meyer and Saedler, 1996). Over the years, it has become clear that HDGS occurs through distinct processes, frequent one being involvement of inverted DNA repeats (IRs) and dsRNA. T-DNA integration at the same chromosomal site leads either to ‘head-to-tail’ direct repeats (DR) or to ‘head-to-head’ or ‘tail to tail’ inverted repeats (IR). T-DNAs that are arranged as IRs are often shown to have low basal expression (Mishiba et al., 2005). IRs have the ability to interact with homologous sequences elsewhere in the genome leading to chromatin remodeling. They can also induce a sequence-specific RNA degradation process, possibly via the formation of dsRNAs (**Figure 1A**).

## Homology Dependent Gene Silencing

A major breakthrough in understanding epigenetic silencing in transgenic plants was first identified in transgenic tobacco, where interaction between two homologous promoters led to DNA methylation and silencing (Matzke et al., 1989). Two types of HDGS are known based on the stage at which it occurs, called transcriptional gene silencing (TGS), which is coupled with transcription or by promoter modification, and post-transcriptional gene silencing (PTGS), which occurs after the formation of mRNA (Jauvion et al., 2012). In TGS, interacting genes that share homology in promoter regions are highly methylated. PTGS involves sequence-specific transcript turnover in the cytosol, which further requires high homology between interacting genes. Potential factors influencing HDGS are degree of homology between the transgene and endogenous gene, the complexity of the host genome, the genomic position of two transgenes, etc. A transgene locus with a complex structure with multiple scrambled T-DNAs has been reported to have strong silencing activities in tobacco, implicating transgene complexity; and vector DNA also decides the efficiency of HDGS (Fu et al., 2000). Complexity of T-DNA structure and integrated vector sequences have been shown to regulate transgene expression in grapevine (Gambino et al., 2010). An increase of endogenous transcript levels above a critical threshold induces specific degradation of homologous transcripts.

## Transcriptional Gene Silencing (TGS)

Transgenes silenced at the transcriptional level acquire metastable epigenetic status that is associated with altered methylation patterns. Transgenes are frequently methylated in cytosine residues that are located within CG, CNN, or CNG sequences. *De novo* DNA methylation can be highly sequence-specific for a specific transgene (Matzke et al., 2007). Fungi or plants expressing foreign genes also exhibit non-symmetrical methylation leading to silencing of endogenous genes. Factors responsible for non-symmetrical methylation are still obscure. Non-symmetrical methylation patterns are aided by RNA- chromatin mechanism (McGinnis et al., 2006).

Methylation in promoter regions, histones, or in coding regions influence gene expression at both the transcriptional (Huettel et al., 2006) and post transcriptional level (Regulski et al., 2013; Tsuchiya and Eulgem, 2013). Chromatin remodeling is involved in maintenance of silenced status and also in transmission of non-symmetrical methylation patterns (Meyer, 1999). Another interesting fact about TGS in transgenic plants is the association of DNA methylation along with structural changes, as methylated and silenced transgenes were less susceptible to endonucleases, reflecting an increased level of chromatin condensation (Van Blokland et al., 1997). Hence, TGS-based silencing might also involve structural changes similar to heterochromatinization, which could be the cause of these structural changes. The responsiveness of TGS of transgenes in response to environmental change was confirmed (Meyer et al., 1992; Meyer, 2015).

Transcriptional gene silencing can be further divided into two classes:

### Transcriptional *cis* Inactivation

In plants, transgenes integrate into the genome at random positions by illegitimate recombination; hence, copy number, their integration site, and local arrangement differ in each transformation event. Also, an inverse relation between transgene copy number and gene expression suggests that multicopy integration can lead to silencing. Integrated foreign genes can undergo TGS in *cis* when multicopy T-DNA is integrated at a locus adjacent to hypermethylated regions of the host genome (Mishiba et al., 2005). More rarely, single copy transgene integration at a hypomethylated locus can lead to *cis* inactivation (Meyer and Heidmann, 1994; Elmayan and Vaucheret, 1996). A maize *A1* gene involved in floral pigmentation when overexpressed in *Petunia* led to silencing of *A1*; however, it was not silenced when *Gerbera dihydroflavonol-4-reductase* was over expressed in *Petunia* suggesting that the transgene also influenced the silencing process. Hence, some degree of difference in DNA composition of the transgene and surrounding host genomic sequences can be recognized by the cellular machinery as foreign non-compatible DNA, leading to specific methylation and silencing (Elomaa et al., 1995). It is believed that *cis* TGS occurs as a result of pairing between closely associated copies of transgenes or endogenous genes, which leads to the formation of secondary DNA structures which are sites for DNA methylation (Vaucheret and Fagard, 2001). Cytosine methylation at CpG and CpNpG sites of transgene and the 35S promoter were also detected in transgenic grapevine transformed with Grapevine fanleaf virus (GFLV) coat protein gene (Gambino et al., 2010).

### Transcriptional Trans-Inactivation

Transcriptional gene silencing can result from unidirectional effects of one transgene on another transgene or homologous endogenous gene. A transgene can be methylated and silenced when it is crossed with a plant in which the homologous gene is in a silenced state (Meyer et al., 1993). *De novo* methylation of one transgene is mediated by a second transgene under control of the same promoter leading to TGS in trans (Fagard and Vaucheret, 2000). Experiments using dsRNA-containing promoter sequences initiated TGS and subsequently *de novo* DNA methylation of the corresponding transgene or endogenous gene, implying a role of an RNA intermediate in TGS (Meyer, 2000). Vaucheret and Fagard (2001) reported the role of different genes, including *ddm1* and *ddm2* in TGS in *Arabidopsis* transgenic lines. Yamasaki et al. (2011) reported methylation of asymmetric cytosine in the enhancer region of 35S promoter in transgenic gentian.

## Post-Transcriptional Gene Silencing

Post-Transcriptional Gene silencing is a condition where transcripts do not accumulate in spite of continuous transcription (Vaucheret et al., 2001). PTGS can silence both transgenes and endogenous genes if both are homologous. An endogenous gene could be switched off, when a plant is transformed with another copy of the same gene. When genes involved in pigmentation, such as *chalcone synthase A* in *Petunia*, were overexpressed, many transgenic lines partially or completely lost activity of both transgene and endogenous gene (Napoli et al., 1990; Van der Krol et al., 1990). This was later called ‘co-suppression’, which was a result of degradation of mRNA of both transgene and endogenous gene. Analysis of degradation products in tobacco expressing β-1,3-glucanase revealed that RNAs are first cleaved by endonucleases, which are further degraded by various exonucleases (Van Eldik et al., 1998). Silencing of two endogenous genes in *Arabidopsis thaliana* was triggered by the antisense and hpRNA transgenes, and silencing in this case was dependent on ploidy level, as it was less pronounced in 4n compared to 2n *Arabidopsis*. Studies indicated that transgenes were more methylated in 4n than 2n *Arabidopsis* suggesting transgenes are transcriptionally repressed in 4n plants, thus resulting in reduced expression levels compared to diploid plants (Finn et al., 2011).

Transgene-induced viral resistance, recovery from infection and proteins encoded by viruses that counteract PTGS suggested it as a potential defense response to check viral infections (Brigneti et al., 1998; Kasschau and Carrington, 1998; Dalmay et al., 2000). It is speculated that the concentration of specific RNAs derived from both transgene and endogenous gene is critical to activate PTGS. dsRNAs are one of the potential candidates, as they are formed between RNAs transcribed from IR and gene homologues. dsRNA is used as a template by RNA-directed RNA polymerase (RdRP) and transcription of dsRNA by RdRP would result in antisense RNAs, which ultimately could target complementary transcripts for degradation by dsRNA-specific RNases (Bond and Baulcombe, 2015; **Figure 1B**).

### Post-Transcriptional *cis*-Inactivation

Post-transcriptional gene silencing *cis*-inactivation is observed when foreign genes like β-Glucuronidase, neomycin phosphotransferase, etc., were driven under strong 35S promoter (Dehio and Schell, 1994; Ingelbrecht et al., 1994; Elmayan and Vaucheret, 1996). When a 35S promoter with a double enhancer was used, more transformants showed PTGS (Elmayan and Vaucheret, 1996; English et al., 1996). Initially, perceptions about PTGS were driven by higher transcript abundance above a threshold level, which ultimately triggered degradation of transgenic RNA. Later, it was found that the level of transcription was not always found to be significantly higher in silenced plants. The presence of IR at transgene locus of silenced lines was proposed to play a crucial role in *cis* inactivation (English et al., 1996). In same year, different models for PTGS were proposed considering RNA abundance and IRs (Baulcombe, 1996). Transgene RNA could be specifically degraded when tagged with specific molecules; these tag molecules were later named small complementary RNA (cRNA). RdRP catalyzed synthesis of cRNA using transgene RNA as template (Dougherty and Parks, 1995). They could also be internal fragments generated from transgene RNA by pairing between aberrant mRNA and normal transgene RNA due to the presence of internal sequence complementarily (Metzlaff et al., 1997). cRNA can interact with mRNA forming dsRNA, which are the target for the cellular enzymes like double-strand RNase. DNA-DNA interactions can lead to methylation, which can further interfere with transcription, ultimately producing aberrant RNA. These aberrant RNAs or higher transcript abundance were owing to the use of a strong promoter that triggered methylation of the coding sequence of the respective transgene (Wassenegger et al., 1994). Interestingly, Kanazawa et al. (2007) reported conversion of PTGS to TGS in *Petunia* transgenic lines as a consequence of the transgene homologous to an endogenous gene in host genome.

### Post-Transcriptional Trans-Inactivation

Post-transcriptional gene silencing was originally reported as coordinated silencing of both transgenes as well as endogenous genes, which is generally termed ‘co-suppression’ (Napoli et al., 1990). Since then, several studies revealed transgenes encoding part of, or the entire transcribed sequence of, host genes have been shown to trigger co-suppression of endogenous genes (Depicker and Van Montagu, 1997). By then it was evident from studies in transgenic *Petunia* lines expressing a chalcone synthase, where efficiency of co-suppression correlated with the strength of the promoter, that there was an effect of transgene dose on co-suppression (Que et al., 1997). Besides, the efficiency of co-suppression is delayed when endogenous host genes are not expressed or when genes are transferred to a mutant devoid of functional gene homologues (Smith et al., 1990; Vaucheret et al., 1997). Hence, it can be concluded that co-suppression cannot be considered as the unidirectional silencing effect of transgenes, rather it is a synergistic phenomenon in which interaction or presence of host genes and transgenes aids aberrant RNA and/or cRNA leading to PTGS.

## Systemic Acquired Silencing

A hallmark of PTGS in plants is that it systemically transmitted in a sequence-specific manner known as systemic acquired silencing (SAS). Remarkable and recurrent features in silencing patterns during developmental stages revealed propagation of a silencing message across the plant (Vaucheret et al., 1998; Kalantidis et al., 2008). Co-suppression of endogenous and transgenes of nitrate reductase, nitrite reductase and SAM synthase in tobacco led to chlorotic or necrotic phenotypes (Boerjan et al., 1994; Palauqui et al., 1996). The non-clonal patterns were observed in all transgenic lines silenced for a specific gene and a sequence-specific message was involved in the control of PTGS. Later, grafting experiments revealed that transgene-specific effector molecules were involved in propagation of *de novo* PTGS over long distances by a phenomenon called SAS (Palauqui et al., 1997). Transgenic tobacco overexpressing *A. thaliana AtMYB90* involved in anthocyanin biosynthesis showed siRNA-mediated silencing as a result of SAS (Velten et al., 2012). A SAS PTGS of transgenes in *N. benthamiana* was initiated in localized regions of the plant when a transgene-homologous DNA was introduced (Voinnet et al., 1998). The silencing signal molecules are degraded RNA, which travels through phloem across cells through plasmodesmata (Kalantidis et al., 2008). The recipient cell can also act as a source for generating secondary signals. It has been reported that sense, antisense, and ill-defined aberrant RNAs can give rise to dsRNA which can transmit signals, ultimately leading to silencing of both transgene and endogenous gene, albeit to different levels (**Figure 1B**).

## Small RNAs as Silencing Signals in Transgenic Plants

RNA was the driving factor for the establishment of DNA methylation patterns (Wassenegger et al., 1994) and acts a signaling agent for inducing silencing. Potato spindle tuber viroid (PSTV) in transgenic tobacco lines led to autonomous viroid RNA replication in the nucleus and induced DNA methylation in the T-DNA (Wassenegger et al., 1994). The evidence from above study clearly indicates the critical role of RNA in initiating *de novo* DNA methylation at homologous regions. Until then, DNA/RNA hybrids were believed to play a role in generating a target for *de novo* methylation. *chsA* co-suppression studies in *Petunia* led to the identification of mobile RNAs as potential candidates responsible for the induction of co-suppression (Napoli et al., 1990; Van der Krol et al., 1990). The initiation of transgene silencing has been thought to involve the generation of dsRNA. It is still under debate about factors triggering initiation of silencing even in the case of transgenes that lack unusual DNA structures.

In plants, micro RNAs (miRNAs) are produced from hairpin-like precursor RNA, which is essential for biogenesis of trans-acting siRNAs (ta-siRNAs). miRNAs are involved in regulation of gene expression by base-pairing with target RNAs further leading to their cleavage in plants. *Physcomitrella patens* transgenic lines expressing different levels of artificial miRNA (amiRNA) revealed transcript-dependent silencing of miRNA target. Thus, a crucial regulatory role of miRNAs might be conserved in other plants also, which are under investigation. siRNAs are another class of small RNAs that are involved in epigenetic modification (Miki and Shimamoto, 2008). Endogenous siRNAs can induce DNA methylation at CpG nucleotides leading to chromatin modification and silencing. Human *H1* and *Arabidopsis 7SL* RNA promoters driving *GUS* specific short hairpin RNA resulted in the efficient silencing of *GUS* at both transcript and protein level, indicating a significant role of siRNAs in epigenetic regulation. However, transgenes are generally more sensitive against RNA silencing than endogenous genes in plants.

## Transgene Silencing as Part of the Host Defense Mechanism?

Silencing cannot be considered as a mechanism that evolved to regulate transgene expression; it is a part of natural plant processes. TGS and PTGS can be considered as host defense responses against ‘foreign invading’ viruses. Hence, transgenes or their products can be equated to cellular invaders triggering defensive reactions leading to silencing of “trans”gene. PTGS recruits cellular components acting against foreign DNA that replicates extra-chromosomally in the nucleus, or RNA in the cytoplasm. A clear connecting link between PTGS and viral resistance was established after the discovery and characterization of various viral proteins that suppress PTGS (Kasschau and Carrington, 1998; Beclin et al., 2002). TGS may use cellular components acting against invading DNA that integrates into the genome. The involvement of DNA methylation can also be considered as a part of cellular defense mechanism against transposable elements. The probable function of dsRNA in initiating methylation can be correlated to retro-elements that produce RNAs with intricate secondary structures.

## Strategies to Prevent Transgene Silencing (Depicker et al., 2005)

(1) Selection of transgenic lines with single T-DNA insert
(2) Organelle targeting/transformation
(3) Selection of favorable/unique integration sites
(4) Reactivation of silent transgenes
(5) *Use of Scaffold Matrix Attachment Regions in silencing mutant host system to prevent silencing*.

## Concluding Remarks

The last three decades have seen immense progress and better understanding of epigenetic effects and silencing mechanisms; transgenic technologies have played a pivotal role for these achievements. Common phenomena behind different types of silencing and recent finding of involvement of siRNAs/miRNA continue to inspire efforts of scientific community to formulate comprehensive models, which also explain the silencing mechanism from an evolutionary view point. Our understanding of the influence of various factors on stability of transgene expression is improving rapidly. We cannot control or predict integration of gene into a recipient genome, nor predict the number of copies or integrity of a transgene. Hence, a comprehensive knowledge of underlying mechanisms in integration process and the influence of chromatin remodeling leading to transgene regulation are crucial. Finally, it might be useful to keep in mind that epigenetic silencing was an unexpected phenomenon; it is still hard to foresee overcoming epigenetic related silencing in transgenic system. Nevertheless, transgenic research will continue as a platform to discover new aspects of epigenetic silencing.

## Author Contributions

SR and PA prepared the manuscript and RS revised it.

## Conflict of Interest Statement

The authors declare that the research was conducted in the absence of any commercial or financial relationships that could be construed as a potential conflict of interest.
